# Multi-Objective Seagull Optimization Algorithm with Deep Learning-Enabled Vulnerability Detection for Secure Cloud Environments

**DOI:** 10.3390/s23239383

**Published:** 2023-11-24

**Authors:** Mohammed Aljebreen, Manal Abdullah Alohali, Hany Mahgoub, Sumayh S. Aljameel, Albandari Alsumayt, Ahmed Sayed

**Affiliations:** 1 Department of Computer Science, Community College, King Saud University, P.O. Box 28095, Riyadh 11437, Saudi Arabia; 2 Department of Information Systems, College of Computer and Information Sciences, Princess Nourah Bint Abdulrahman University, P.O. Box 84428, Riyadh 11671, Saudi Arabia; 3 Department of Computer Science, College of Science & Art at Mahayil, King Khalid University, Abha 61413, Saudi Arabia; 4 SAUDI ARAMCO Cybersecurity Chair, Department of Computer Science, College of Computer Science and Information Technology, Imam Abdulrahman Bin Faisal University, P.O. Box 1982, Dammam 31441, Saudi Arabia; 5 Department of Computer Science, Applied College, Imam Abdulrahman Bin Faisal University, P.O. Box 1982, Dammam 31441, Saudi Arabia; afaalsumayt@iau.edu.sa; 6 Research Center, Future University in Egypt, New Cairo 11835, Egypt; a.sayed@fueinstitute.net

**Keywords:** cloud computing, deep learning, intrusion detection system, sooty tern optimization algorithm, seagull optimization algorithm

## Abstract

Cloud computing (CC) is an internet-enabled environment that provides computing services such as networking, databases, and servers to clients and organizations in a cost-effective manner. Despite the benefits rendered by CC, its security remains a prominent concern to overcome. An intrusion detection system (IDS) is generally used to detect both normal and anomalous behavior in networks. The design of IDS using a machine learning (ML) technique comprises a series of methods that can learn patterns from data and forecast the outcomes consequently. In this background, the current study designs a novel multi-objective seagull optimization algorithm with a deep learning-enabled vulnerability detection (MOSOA-DLVD) technique to secure the cloud platform. The MOSOA-DLVD technique uses the feature selection (FS) method and hyperparameter tuning strategy to identify the presence of vulnerabilities or attacks in the cloud infrastructure. Primarily, the FS method is implemented using the MOSOA technique. Furthermore, the MOSOA-DLVD technique uses a deep belief network (DBN) method for intrusion detection and its classification. In order to improve the detection outcomes of the DBN algorithm, the sooty tern optimization algorithm (STOA) is applied for the hyperparameter tuning process. The performance of the proposed MOSOA-DLVD system was validated with extensive simulations upon a benchmark IDS dataset. The improved intrusion detection results of the MOSOA-DLVD approach with a maximum accuracy of 99.34% establish the proficiency of the model compared with recent methods.

## 1. Introduction

Cloud computing (CC) offers numerous services to users including infrastructure, storage capabilities, and applications [1]. A cloud user can manipulate or access software and hardware over the internet based on their requirements. Though CC provides several advantages to its users, it also has certain limitations and challenges. These challenges include performance management, privacy, security, cost, and load balance [2]. Among the issues encountered by the cloud computing phenomenon, security plays a major role in user data and applications on the cloud infrastructure. CC security encompasses policies and procedures to protect cloud-based information, applications, and frameworks from unauthorized access and attacks [3]. Also, it protects data and infrastructure against Structured Query Language (SQL) injection, software vulnerability, flooding attacks, cross-site scripting, data alteration, and data leakage. In parallel, cloud providers and subscribers continuously report security problems raised by different types of attacks. Hence, it is necessary to provide security against malicious activities and attacks [4].

Intrusion detection systems (IDSs) [5] in cloud networks play a crucial role in terms of providing security against attacks from both outsiders as well as insiders [6]. Traditional IDSs are used in the detection of attacks in internet environments. However, they cannot adjust their working mechanisms for cloud platforms and so remain non-scalable. Furthermore, researchers found them to be not appropriate for cloud platforms and not deterministic [7]. Therefore, new and reliable anomaly based IDSs have been proposed, developed, and validated. Mostly, the existing methods for anomaly detection from cloud platforms used machine learning (ML) approaches. These methods can enhance their performance by upgrading their data according to the pattern detected from the input datasets [8]. When a novel pattern is detected from the input dataset, the ML technique parameters are upgraded for the detection of the same anomalies in future traffic flow. According to the data extracted from the prior outcomes, the solution of the method is enhanced by altering the implementation approach, if required. The feature selection (FS) process helps to focus only on the most related information. FS is an ML method that reduces the quantity of the data to be analyzed [9]. It can be achieved by detecting the relevant features (such as the attributes) of a dataset, leaving behind the insignificant ones. By reducing the dimensionality of a dataset, i.e., retaining only the relevant features, the ML technique can make the classification prediction process an efficient and effective one [10]. This efficacy is particularly related to the intrusion detection (ID) process that needs real-time performance.

The current study designs a new multi-objective seagull optimization algorithm with a deep learning-enabled vulnerability detection (MOSOA-DLVD) system for a secure cloud platform. In the developed MOSOA-DLVD algorithm, the feature selection process is performed with the help of the MOSOA technique. Furthermore, the MOSOA-DLVD technique uses a deep belief network (DBN) method for intrusion detection and classification. To enhance the detection results of the DBN algorithm, the sooty tern optimization algorithm (STOA) is implemented for the hyperparameter tuning process. The performance of the MOSOA-DLVD system is examined with simulations using a benchmark IDS database. The main contributions of the current study are briefly given below.

- Development of an automated intrusion detection system for the cloud platform, named the MOSOA-DLVD algorithm, which involves MOSA-based FS, DBN-based classification, and STOA-related hyperparameter tuning. To the best of the authors’ knowledge, the MOSOA-DLVD system was previously non-existent in the literature.
- The development of the MOSOA approach supports the selection of related features, increases accuracy, and reduces higher dimensionality issues.
- Hyperparameter tuning of the DBN model, using the STOA, enhances the prediction outcomes of the MOSOA-DLVD algorithm for hidden data.

The remaining sections of this paper are explained here. Section 2 offers the related works, and Section 3 provides details about the developed model. Next, Section 4 discusses the outcomes of the analyses, and Section 5 concludes this paper.

## 2. Related Works

Kavitha et al. [11] examined filter-based ensemble-FS (FEFS) and used the DL method to overcome the problems faced in CC. FEFS is an integration of three feature extraction approaches, namely, embedded, filter, and wrapper methods. In these feature extraction models, the important features were selected to enable the trained model in the DL technique. Lastly, the classifier accomplished the FS. The DL method was an integration of two techniques including the Tasmanian devil optimization (TDO) and the recurrent neural network (RNN). The authors [12] developed an innovative IDS, which incorporates the fuzzy C-means (FCM) technique with SVM to improve the accuracy of the recognition systems at CC platforms. Maheswari et al. [13] suggested a hybrid soft computing-assisted IDS, i.e., ST-IDS for cloud and web platforms. The authors proposed an IDS system for CC and web infrastructure by utilizing the hybrid teacher learning-enabled-DRNN (TL-DRNN) and cluster-related feature optimizer. In their study, the modified manta ray foraging optimizer (MMFO) was used after feature extraction in the selection of optimum features for accurate detection. The hybrid TL-DRNN was devised to classify the intrusions from the web and cloud platforms. In [14], the authors proposed a dual-channel capsule generative adversarial network (GAN) optimized with RFO algorithm-fostered IDS (IDS-CC-DCCGAN-RFOA) to ensure privacy and secure the CC platform from different types of attacks. According to the best features, the data were categorized into two models, namely, privacy attack and secured data, depending on the DCCGAN outcome. Then, the weight of the DCCGAN model was optimally fine-tuned utilizing the RFO method to accomplish the efficient and best outcomes in terms of intrusion detection. 

In a study conducted earlier [15], the authors developed the LR-based oppositional tunicate FCM (LR-OTSFCM) method for cloud ID. The important part of this study is the identification of the attacks in the cloud platform. In [16], a novel hybridization approach was suggested for the IDS to enhance the complete security of the cloud-based computing platforms. In addition, the SMO technique was also used in that study to reduce the dimensionality reduction. The datasets were fed into a neural network (NN). The authors [17] recommended the efficient dragonfly-improved invasive weed optimizer-assisted Shepard-CNN (DIIWO-based ShCNN) technique for identifying the attackers and alleviating the attacks in the cloud model. It is highly possible for the model to detect intruders with ShCNN. In [18], an efficient IDS, termed the chronological salp swarm algorithm-based DL model, was designed to identify suspicious intrusions in the cloud platform. The presented method was developed by combining the chronological idea and SSA. The optimum solution to detect the intrusion was exposed by utilizing the fitness function (FF), which considers the minimum error value as the optimal result. In a study conducted earlier [19], a novel design for deep LSTM-based IDS was presented for detecting the network traffic flow designs from the cloud platform and distinguishing them as malicious or normal patterns. The presented IPS avoids the malicious attacks received in the IDS by improving the recognition rate of the malicious attacks and reducing the computational time. The DNN with game theory for cloud security (GT-CSDNN) model was presented in a study conducted earlier [20]. The developed model covered either attacker or defender approaches but used the game theory algorithm. Furthermore, the DNN model utilized the presented game theory approach for classifying the attacks from regular data. In [21], a new ML-based hybrid IDS was presented. In that study, the integrated SVM and GA approach was established with a novel FF to evaluate the accuracy of the system.

Alohali et al. [22] presented the improved metaheuristics with a fuzzy logic-based intrusion detection system for cloud security (IMFL-IDSCS) technique. For their study, an individual IDS sample was deployed, and the IMFL-IDSCS technique used the enhanced chimp optimization algorithm-based feature selection (ECOA-FS) method for the selection of the optimal features, followed by the adaptive neuro-fuzzy inference system (ANFIS) model. In a study conducted earlier [23], the authors suggested a novel IDS by combining leader-based K-means clustering (LKM) and an optimal fuzzy logic system. Initially, the input dataset was grouped into clusters using the LKM technique. Then, the cluster data were fed into the fuzzy logic system (FLS). Both normal and abnormal data were inquired by the FLS, whereas the FLS was trained with the grey wolf optimization algorithm by maximizing the rules. Mahmood et al. [24] proposed an approach for obtaining the optimal number of features so as to build an efficient IDS model. In their study, feature reduction was applied. Generalization ability can be improved in general by generating a small set of features from the actual input variables using feature extraction. For their study, a hybrid algorithm, named the principal component analysis neural network algorithm (PCANNA), was used to reduce the number of computer resources.

Although several studies have been conducted for intrusion detection in the cloud platform, the prominence of the FS with hyperparameter tuning for differentiating attacked traffic from normal traffic is yet to be completely studied. Though the implementation of the ML-based IDS was developed earlier, the unique dynamics of the cloud platforms, represented by its various and dynamic workloads, demand specified methods. The existing research shortages drive the demand for a comprehensive scheme that can select important and essential features from the massive quantity of accessible data in order to increase the proficiency and performance of the intrusion detection process. On the other hand, fine-tuning the hyperparameters is frequently disregarded, which in turn results in sub-optimum model effectiveness. Additionally, the important aids of ensemble learning, in which many detection frameworks are incorporated to use their collected predictive capability, are not progressively combined into the ID pipeline. To overcome this research gap, it is vital to design a highly robust and effective intrusion detection technique that is customized according to the particular challenges, modeled with cloud platforms. This way, it becomes possible to finally improve their security posture and alleviate the development of threats. So, it is essential to enhance the generalizability, robustness, and accuracy of the intrusion detection methods, mainly in dynamic and developing network infrastructures. However, the attacks endure to develop in such sophistication and complication as well. Both FS and hyperparameter tuning include various search spaces. FS normally contains a discrete search space, whereas various integrations of the features are estimated. Alternatively, hyperparameter tuning often comprises semi-continuous or continuous search spaces for parameter values. The contribution of MOSOA for FS and STOA, in terms of hyperparameter tuning, allows every method to consider its corresponding search space and the multiplication of its efficacy and performance. MOSOA was developed for multi-objective optimizer tasks, which makes it a well-suitable FS. However, the aim is to enhance numerous conflicting criteria, namely, interpretability, accuracy, and dimensionality reduction. On the contrary, STOA can be highly proficient at enhancing hyperparameters because of its unique optimization approaches.

## 3. The Proposed Model

**Figure 1:**
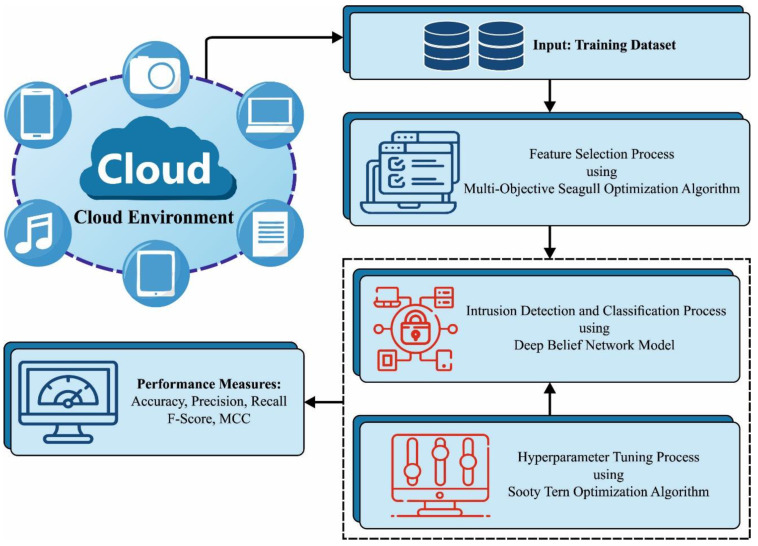
The overall flow of the MOSOA-DLVD algorithm.

In the current study, the authors designed the MOSOA-DLVD methodology for accomplishing security in the cloud platform. The aim of the MOSOA-DLVD algorithm is to identify the presence of vulnerabilities or attacks in the cloud platform. The model has three phases of function: the MOSOA-based FS, DBN classification, and STOA-based hyperparameter selection. Figure 1 exemplifies the workflow of the MOSOA-DLVD method.

### 3.1. Feature Selection Using MOSOA

The MOSOA technique is used to select the better feature sets. This technique is imitated for the process of FS in which seagulls function as searching agents (features) [25]. SOA is a meta-heuristic optimizer algorithm inspired by the foraging behavior of seagulls. This algorithm provides the benefits of a modest implementation and architecture. The major benefit of the SOA is that its overall construction and composition are relatively simple, while its global search and local search abilities are strong. Here, the migration method is performed to attain the optimum features out of an accessible group of features and to explore the search space. The main function of the FS method includes a reduction in classification errors and the features that are considered as input.

(1) $$Min\;F_{t}=\delta^{*}\Psi +(1-\delta {)}^{*}\frac{\left|f\right|}{\left|F\right|}\;\;$$

In this system, the aims are combined into a single objective equation like a preset weight that identifies all the objective importance. In Equation (1), δ corresponds to the parameter inducing the classifier’s output, F denotes the overall number of features in the data, Ψ specifies the error rate of the classifiers, and f represents the overall feature extraction counts during the extraction feature. The FF needs to have a low value for the proper FS.

Exploration: The exploration of the search agent includes its movement from one place to another as per the FF. The three most important conditions of the exploration method of MOSOA are given below:

(i). Collision Avoidance: It is also possible for a collision to happen, so a parameter is used to calculate the location of the searching agent while exploring the search range. The equation is given below.

(2) $${\vec{c}}_{s}=A^{*}{\vec{p}}_{s}\left(x\right)\;$$

In Equation (2), ${\vec{c}}_{s}$ shows the location of the searching agent not included in a collision, ${\vec{p}}_{s}$ represents the existing location of the searching agent, x denotes the present iteration, and parameter A shows the movement of the searching agent from the performance space. The formula for the parameter is given below.

(3) $$A=f_{\iota }-\left(x^{*}\left(\frac{f,}{Itr_{max}}\right)\right);x=0,1,\ldots Itr_{max}\;\;\;\;\;\;$$

In Equation (3), f controls the frequency of the A parameter.

The movement to the optimum neighbor location: The searching agent that avoids the collision moves to a better neighborhood position, for which the formula is as follows.

(4) $${\vec{m}}_{s}=B^{*}\left({\vec{p}}_{bs}\left(x\right)-{\vec{p}}_{s}\left(x\right)\right)\;\;\;\;$$

In Equation (4), ${\vec{p}}_{s}$ corresponds to the searching agent, ${\vec{p}}_{bs}$ stands for the place of the better search agent, and ${\vec{m}}_{s}$ represents the movement of ${\vec{p}}_{s}$ toward ${\vec{p}}_{bs}$. The random value B is accountable for maintaining the balance between the exploitation and exploration phases. The formula for B is given below.

(5) $$B=2^{*}A^{2_{*}}rnd$$

In Equation (5), rnd denotes a random value within [0, 1].

(ii). Position Update: Finally, the searching agent updates the location based on the location of a better searching agent in the group. The location updating formula is as follows.

(6) $${\vec{d}}_{s}=\left|{\vec{c}}_{s}+{\vec{m}}_{s}\right|\;\;\;$$

In Equation (6), ${\vec{d}}_{s}$ denotes the distance between the better one in the group and the searching agent.

The MOSOA technique calculates the fitness function of the searching agent, whereas a better solution is upgraded to the archive. Once the archive is established to overflow, the grid technique is used to avoid the crowded solution in the available solutions from the archive. Next, a novel solution is upgraded to archive and later, the boundary of the searching agent is adjusted and evaluated. Finally, the FF estimates the position of the searching agent in the archive, whereas the better searching agent is upgraded with a novel location.

Exploitation: This procedure is imitated during the attacking behavior of the searching agent based on the experience and history of the exploitation. The searching agent spirally moves from the air in a 3D axis and is defined as follows.

(7) $$x^{\prime }=\alpha^{*}cos\left(l\right)\;$$

(8) $$y^{\prime }=\alpha^{*}sin\left(l\right)\;$$

(9) $$z^{\prime }=\alpha^{*}l\;\;\;$$

(10) $$l=u^{*}e^{lv}$$

where α represents the radius of each turn in a spiral movement, *l* denotes the arbitrary value selected in the range of [0, 2π], and u and v are the constants that represent the spiral motion. The last upgraded location of the search agent is shown below.

(11) $${\vec{p}}_{s}\left(x\right)=\left({{\vec{d}}_{s}}^{\;}*x^{\prime }*y^{\prime }*z^{\prime }\right)+{\vec{p}}_{bs}\left(x\right)$$

In the MOSOA technique, the better Pareto optimum result is compared with that of the current solution. Therefore, this method selects the leader for the group to achieve it. The minimum crowded space from the archive is occupied with the roulette wheel selection process, whereas the better solution in the optimum boundary is taken into account as given below.

(12) $$U_{l}=\frac{h}{N_{l}}$$

In Equation (12), $N_{l}$ shows the amount of Pareto optimum solutions for the segment and h denotes the constant value higher than *l*.

### 3.2. Vulnerability Detection Utilizing the DBN Model

The DBN model has been applied in the detection and classification of the vulnerabilities. DBNs can automatically learn the hierarchical representations of the input data. For the purpose of intrusion detection, it is used for learning and extracting the important features from raw network traffic data and reducing the requirement for manual feature engineering. Primarily, this characteristic is valuable for a network intrusion model as it is complex and develops over some time. DBNs have been well-appropriated for anomaly detection activity, which is an important module of the intrusion detection process. It can model the normal behavior of a network and indicate abnormalities from learned regularities as possible intrusions. It is supported to identify new or earlier hidden attack patterns. It is capable of taking reliance and correlation among the diverse phases of multi-stage attacks. Generally, this is significant as advanced attacks include several stages, and identifying them as a whole could be more efficient than detecting different types of anomalies.

**Figure 2:**
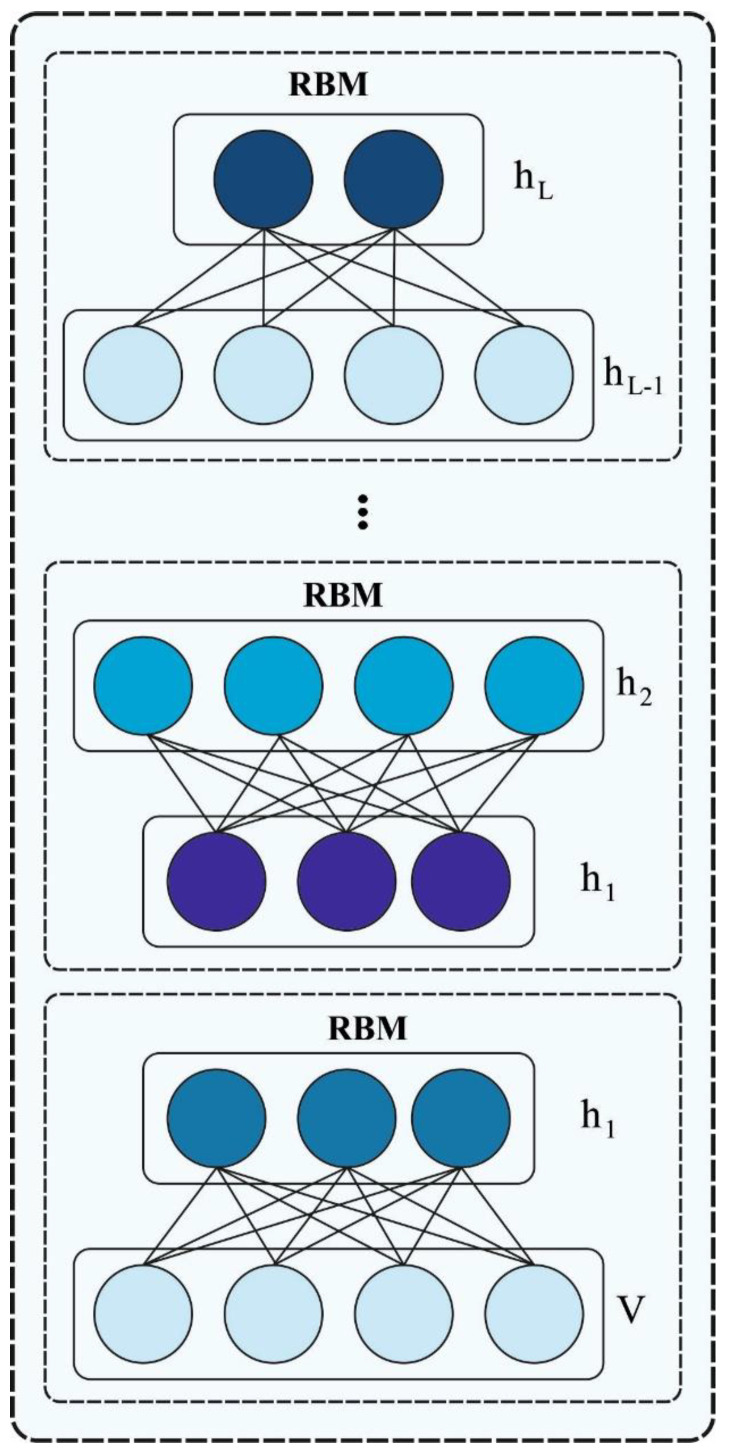
DBN structure.

DBN is considered a fusion of unsupervised network models like RBM that act as a hidden layer (HL) of each subnet and a visible layer (VL) of the second layer [26]. The DBN model comprises multiple VLs, HLs, and an LR for classification in the final layer. Initially, the feature vector is mapped, after which, each layer of the RBM is trained using an unsupervised method for maintaining the feature data. Next, a fine adjustment is made. In the RBM technique, the $v_{i}$ in the VL and HL are characterized as $h_{i}$. $w_{ij}$ represents the weights between $v_{i}$ and $h_{j},$ while the latter denotes the guided values. The VL and HL nodes have biases and are denoted by the c and b vectors. The $b_{i},$ $c_{i}$, and $w_{ij}$ values of the RBM form the parameter θ in the DBN and appear in the model with a probability of the energy function and the HL. Figure 2 represents the framework of the DBN.

$$E\left(\theta ,v,h\right)=-\sum_{i=1}^{m}v_{i}c_{i}-\sum_{j=1}^{n}h_{j}b_{j}$$

(13) $$-\sum_{i=1}^{m}\sum_{j=1}^{n}v_{i}h_{i}w_{ij}\;\;\;\;\;$$

Subsequently, there is no interlayer linked from the DBN model, whereas the probability distribution of the VL and HL is computed as given below.

(14) $$P\left(v_{i}=1|h\right)=1/1+e^{-b_{i}\Sigma h_{i}w_{ij}}\;\;\;\;\;\;\;$$

(15) $$P\left(h_{i}=1|v\right)=1/1+e^{-c_{i}\Sigma v_{i}w_{ij}}\;\;$$

The reconstructed data are returned and defined with the $p\left(vh\right)$ computation after the weight calculation is completed. The output σ takes place once the data are transferred back to the HL. Now, the logistic function σ can be described as follows.

(16) $$\sigma (x)=(1+e^{-x}{)}^{-1}$$

Similarly, if $v_{i}=1$, the conditional probability of $v_{i}$ can be computed as follows:

(17) $$P\left(v_{i}=1|v\right)=\sigma \left(a_{i}+\sum_{i=1}W_{ij}h_{j}\right)$$

### 3.3. Hyperparameter Tuning Using the STOA

Eventually, the STOA is utilized for the optimum hyperparameter selection of the DBN approach. The STOA is a new optimization technique derived from the natural foraging behavior of seabirds [27]. The sooty tern is an omnivorous bird that preys on fish, earthworms, and other insects. The technique has high precision and a strong global search ability. The STOA can be a population-based technique separated into local and global search phases. The global search phase mainly comprises collision avoidance, position update, and convergence to the optimum solution.

(1) The mathematical equation is used for collision avoidance is as follows.

(18) $$B=\gamma \times P\left(k\right)$$

(19) $$\gamma =\alpha -\left(k\times \left(\alpha -{Max}_{iieraiion}\right)\right)k=0,1,2,\;\cdots {Max}_{iteration},\;\;\;\;\;\;\;$$

where B refers to the safer location to make sure that no collision occurs between the black terns; γ denotes the collision avoidance aspect; and P(k) shows the existing location of the black tern. k represents the number of iterations; and the α value is 2.

(2) Convergence to the optimum solution is formulated as follows.

(20) $$\left\{\begin{array}{l} M=\beta \times \left(P_{b}\left(k\right)-P\left(k\right)\right)\\ \beta =0.5\times r \end{array}\right.$$

In Equation (20),  p(k) shows the existing optimum tern, M denotes the optimum location of the sooty tern colony; β refers to the arbitrary regulator; and r is an arbitrary integer in the range of [0, 1].

(3) To update the position, the following equation is used.

(21) D=B+M

In Equation (21), D denotes the existing and optimum locations of a sooty tern.

During the local exploration stage, the bird uses its wings to gain height and also changes its angle and speed of attack during the migration process. The hovering behavior at the time of attacking prey is described as follows.

(22) $$\left\{\begin{array}{l} x^{\prime }=R\times cos(\theta )\\ y^{\prime }=R\times cos(\theta )\\ z^{\prime }=R\times \theta \\ r=u\times e^{kv} \end{array}\right.$$

In Equation (11), θ represents the angle of attack in the range of $\left[0,2\right]$, R denotes the spiral radius, and u and v show the spiral constant and are fixed as 1. The equation to update the location of the sooty tern is as follows.

(23) $$P\left(k\right)=\left(D\times \left(x^{\prime }\times y^{\prime }\times z^{\prime }\right)\right)\times P_{b}\left(k\right)$$

FF is a key feature of the STOA system. The encoder performance is used to develop the optimum candidate outcome. Presently, accuracy is the main condition deployed to develop the FF.

(24) $$Fitness=max\left(\frac{TP}{TP+FP}\right)$$

where TP and FP stand for true and false positive values, respectively.

## 4. Results and Discussion

The MOSOA-DLVD methodology was experimentally validated using the NSL-KDD database [28]. The dataset has a total of 125,973 samples under five classes, as shown in Table 1.

**Figure 3:**
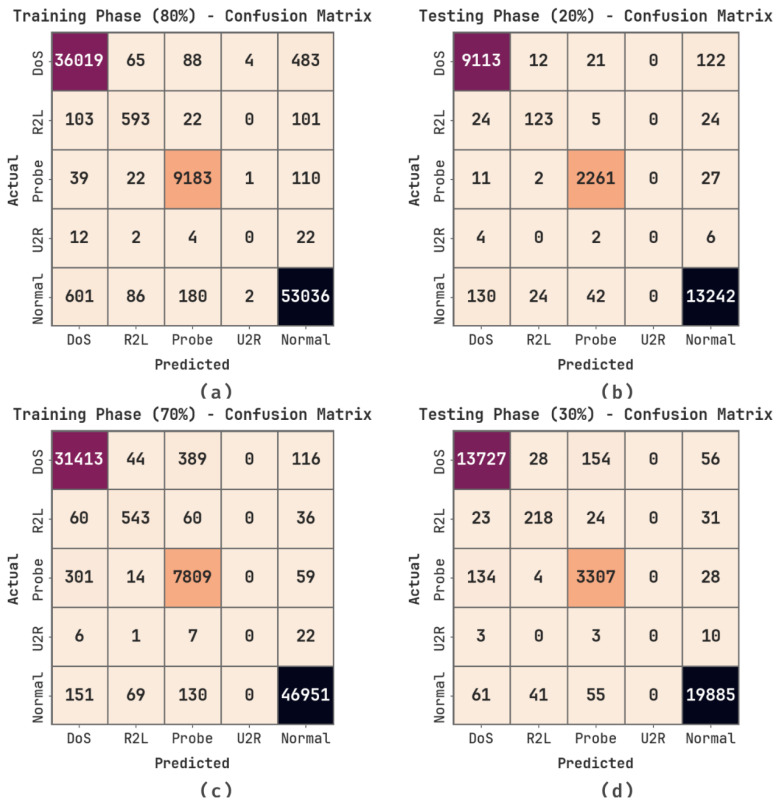
(**a**,**b**) Confusion matrices at 80:20 of TRS/TSS and (**c**,**d**) 70:30 of TRS/TSS.

In Figure 3, the confusion matrices generated using the MOSOA-DLVD system are shown. The outcomes indicate that the MOSOA-DLVD algorithm accurately recognized all five classes.

**Figure 4:**
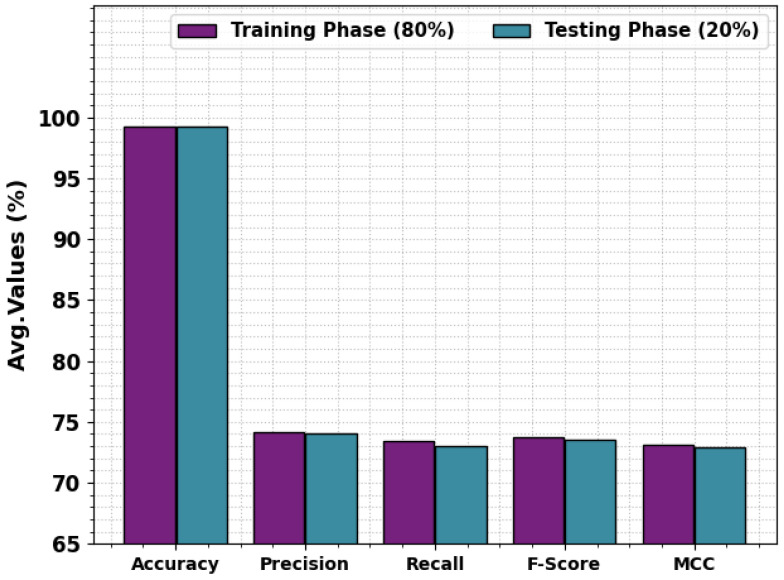
Average analysis outcomes of the MOSOA-DLVD model with 80% of TRS/20% of TSS.

In Table 2 and Figure 4, the overall detection results of the MOSOA-DLVD method at 80:20 of the TRS/TSS are given. The achieved outcomes show that the MOSOA-DLVD system proficiently recognized all five class labels. At 80% of the TRS, the MOSOA-DLVD algorithm achieved an average $accu_{y}$ of 99.23%, $prec_{n}$ of 74.15%, $reca_{l}$ of 73.44%, $F_{score}$ of 73.78%, and an MCC of 73.14%. Next, with 20% of the TSS, the MOSOA-DLVD system obtained an average $accu_{y}$ of 99.28%, $prec_{n}$ of 74.05%, $reca_{l}$ of 73%, $F_{score}$ of 73.50%, and an MCC of 72.90%.

**Figure 5:**
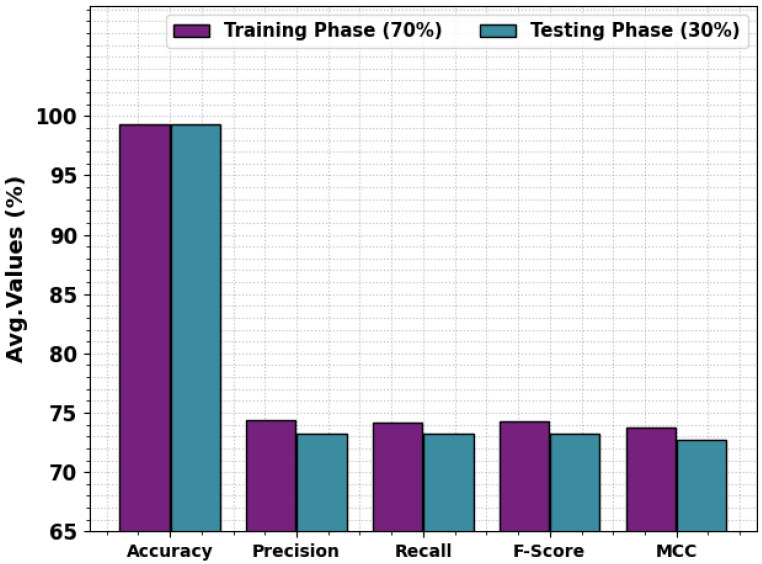
Average analysis outcomes of the MOSOA-DLVD method with 70% of TRS/30% of TSS.

The overall detection outcomes of the MOSOA-DLVD algorithm at 70:30 of TRS/TSS are portrayed in Table 3 and Figure 5. The outcomes illustrate that the MOSOA-DLVD method efficiently recognized all five classes. For 70% of the TRS, the MOSOA-DLVD methodology attained an average $accu_{y}$ of 99.34%, $prec_{n}$ of 74.37%, $reca_{l}$ of 74.13%, $F_{score}$ of 74.24%, and an MCC of 73.76%. With 30% of the TSS, the MOSOA-DLVD system attained an average $accu_{y}$ of 99.31%, $prec_{n}$ of 73.21%, $reca_{l}$ of 73.28%, $F_{score}$ of 73.24%, and an MCC of 72.73%.

**Figure 6:**
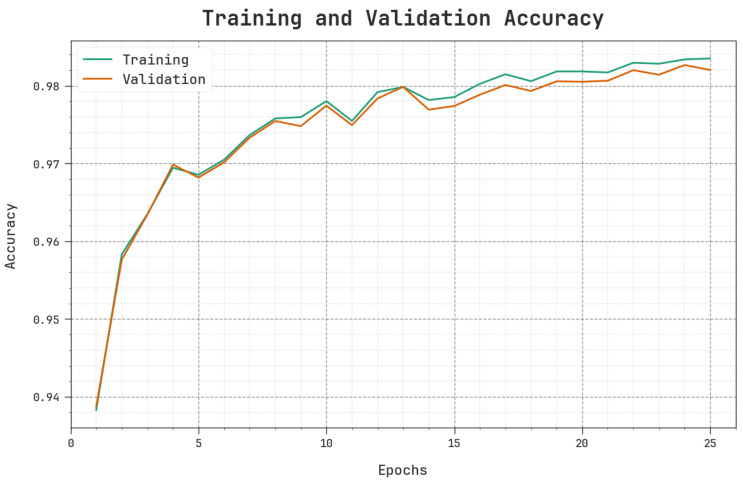
$Accu_{y}$ curve of the MOSOA-DLVD algorithm.

Figure 6 represents the training accuracy $TR\_accu_{y}$ and $VL\_accu_{y}$ values attained with the MOSOA-DLVD algorithm. $TL\_accu_{y}$ is determined by validating the MOSOA-DLVD methodology using the TR database, whereas $VL\_accu_{y}$ is measured as the effectiveness of the model upon a distinct TS dataset. The results show that the $TR\_accu_{y}$ and $VL\_accu_{y}$ values increase with an increase in the number of epochs. Accordingly, the effectiveness of the MOSOA-DLVD algorithm is enriched with the TS and TR datasets. 

**Figure 7:**
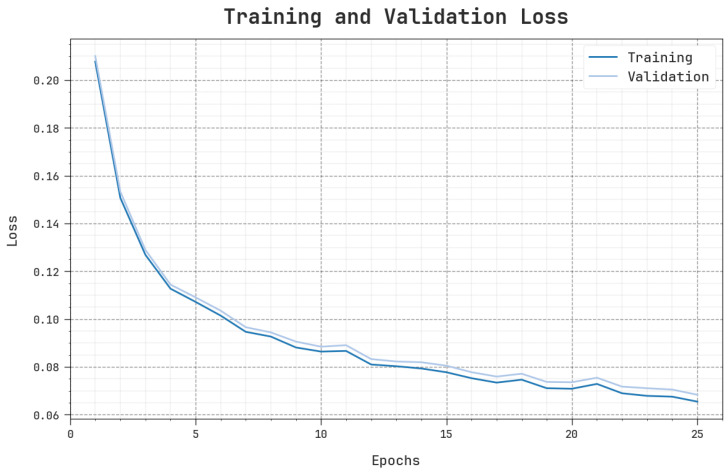
Loss curve of the MOSOA-DLVD system.

In Figure 7, the TR_loss and VR_loss  curves of the MOSOA-DLVD methodology are illustrated. TR_loss corresponds to the errors between the original and the predicted values in the TR data. VR_loss denotes the measurement of the MOSOA-DLVD system on specific validation data. The obtained outcomes confirm that both TR_loss and VR_loss values are reduced with an increasing number of epochs. This outcome describes the enriched effectiveness of the MOSOA-DLVD approach as well as its ability to achieve accurate classification. The minimal TR_loss and VR_loss values reveal the superior performance of the MOSOA-DLVD algorithm on correlation and capturing patterns.

**Figure 8:**
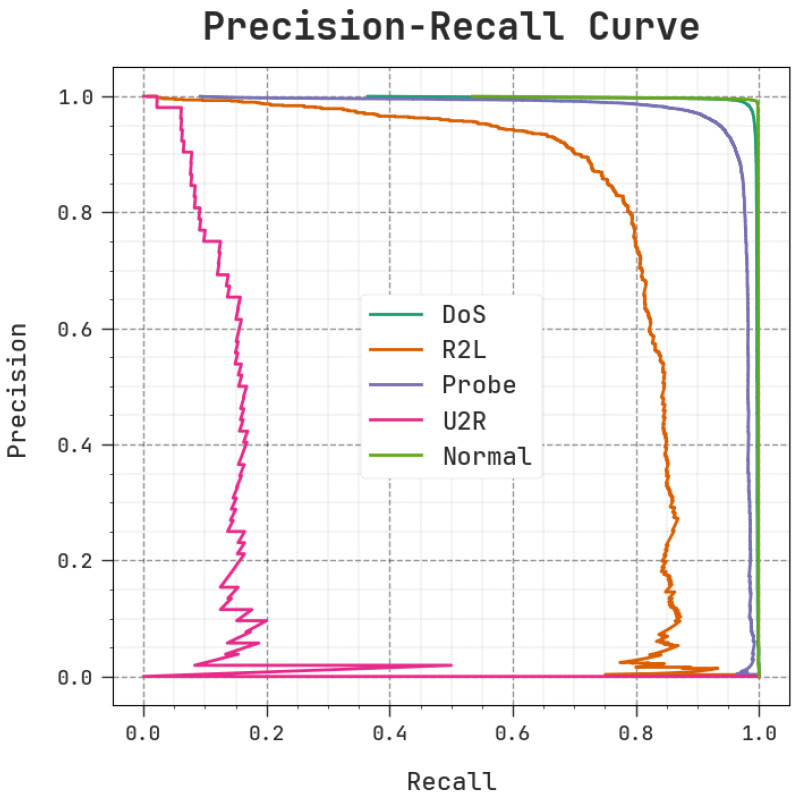
PR analysis of the MOSOA-DLVD model.

The comprehensive precision–recall examination outcomes produced using the MOSOA-DLVD approach with the test dataset are shown in Figure 8. The MOSOA-DLVD algorithm was found to achieve increased PR values. In addition, it is obvious that the MOSOA-DLVD algorithm attains superior precision–recall values for all five classes.

**Figure 9:**
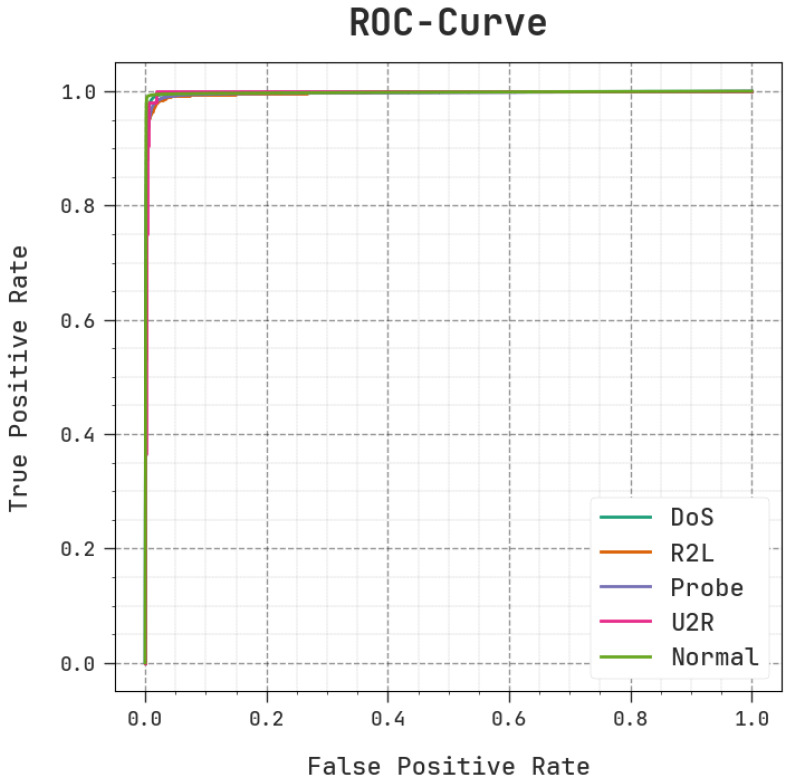
ROC curve of the MOSOA-DLVD algorithm.

In Figure 9, the ROC outcomes of the MOSOA-DLVD methodology are exhibited. The outcomes show that the MOSOA-DLVD system produced enhanced ROC values. Furthermore, it is apparent that the MOSOA-DLVD algorithm extends greater ROC values with all five classes. The ROC curves produced using the MOSOA-DLVD system exhibit its capability to differentiate the classes. This figure indicates the valued perceptions of the trade-off between the FPR and TPR rates over individual categorization thresholds as well as the changing number of epochs. This figure displays the predicted $accu_{y}$ efficiency of the MOSOA-DLVD model for the categorization of diverse classes.

**Figure 10:**
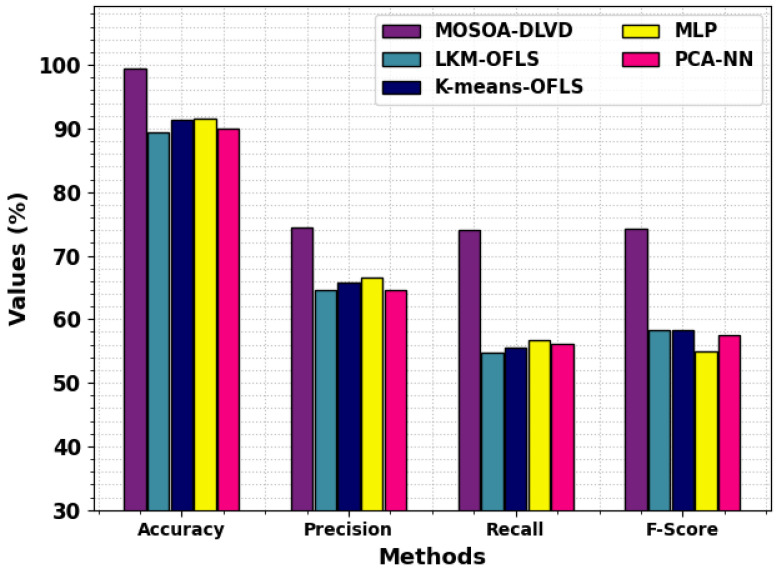
Comparative analysis of the outcomes of the MOSOA-DLVD algorithm and other systems.

A comparison analysis was conducted between the MOSOA-DLVD methodology and other existing systems such as the leader-based K-means clustering (LKM) with the OFLS [22], K-means with OFLS [23], MLP [23], and PCA with NN [24] methods, and the results are portrayed in Table 4 and Figure 10 [22,23,24]. The achieved outcomes show that the LKM-OFLS and PCA-NN models obtained poorer results than the rest of the models. Along with that, the K-means-OFLS and MLP techniques accomplished a closer performance. But the MOSOA-DLVD technique reported the maximum performance with $accu_{y}$, $prec_{n}$, $reca_{l}$, and $F_{score}$ values being 99.34%, 74.37%, 74.13%, and 74.24%, respectively. This phenomenal performance establishes the enhanced outcomes of the MOSOA-DLVD methodology.

In summary, the MOSOA-DLVD method exhibited superior performance with a maximum accu_y of 99.34%. The high effectiveness of the MOSOA-DLVD system is due to the incorporation of the MOSOA-assisted FS algorithm and STOA-based hyperparameter tuning. The MOSOA algorithm selects the relevant and beneficial features at accessible feature sets. With the elimination of unrelated features, the proposed model can be considered a crucial finding in terms of aspects contributing to the classification method. This model can improve the accuracy of classification. Alternatively, the STOA optimizer prefers the optimal values for the hyperparameters of the specified DBN system. If the hyperparameters cannot be learned during the training period, then they should be set before the training. It has an important effect on the model’s performance as well, and the selection of the optimum values could result in higher accuracy. By integrating the MOSOA-based FS algorithm and STOA-based hyperparameter tuning, the MOSOA-DLVD system achieved the best solution by emphasizing major related features as well as selecting the optimal sets for the method. These attained outcomes confirm the better performance of the MOSOA-DLVD methodology over other systems.

## 5. Conclusions

In the current study, the MOSOA-DLVD technique was presented to accomplish security in the cloud platform. The primary aim of the MOSOA-DLVD methodology is to identify the presence of vulnerabilities or attacks in the cloud platform. In the developed MOSOA-DLVD method, three phases of processes are executed such as the DBN classification, STOA-based hyperparameter selection, and the MOSOA-based FS. To enhance the detection results of the DBN algorithm, the STOA was used for hyperparameter tuning. The performance of the MOSOA-DLVD method was examined using the benchmark NSL-KDD dataset. A wide range of simulations was conducted, and the outcomes established the improved intrusion detection outcomes of the MOSOA-DLVD system over existing methodologies with a higher accuracy of 99.34%. In the future, the MOSOA-DLVD method can be extended to the big data environment. Furthermore, the class imbalance data handling issue needs to be resolved in order to achieve improved classification results. Future works can explore further techniques for intrusion detection that can operate on encrypted or privacy-preserving data. This might ensure the confidentiality of sensitive information, while detecting intrusions in an effective manner and remains important, especially in multi-tenant cloud environments.

## Figures and Tables

**Table 1:** Description of the dataset.

Class	No. of Samples
Dos	45,927
R2l	995
Probe	11,656
U2r	52
Normal	67,343
Total no. of Samples	125,973

**Table 2:** Detection outcomes of the MOSOA-DLVD algorithm on 80% of TRS/20% of TSS.

Labels	$Accu_{y}$	$Prec_{n}$	$Reca_{l}$	$F_{Score}$	MCC
TSR (80%)					
DoS	98.62	97.95	98.25	98.10	97.01
R2L	99.60	77.21	72.41	74.73	74.57
Probe	99.54	96.90	98.16	97.53	97.27
U2R	99.95	00.00	00.00	00.00	-0.02
Normal	98.43	98.67	98.39	98.53	96.84
Average	99.23	74.15	73.44	73.78	73.14
TSS (20%)					
DoS	98.71	98.18	98.33	98.25	97.24
R2L	99.64	76.40	69.89	73.00	72.89
Probe	99.56	97.00	98.26	97.63	97.39
U2R	99.95	00.00	00.00	00.00	00.00
Normal	98.51	98.67	98.54	98.60	97.01
Average	99.28	74.05	73.00	73.50	72.90

**Table 3:** Detection outcomes of the MOSOA-DLVD algorithm on 70% of TRS/30% of TSS.

Labels	$Accu_{y}$	$Prec_{n}$	$Reca_{l}$	$F_{Score}$	MCC
TRS (70%)					
DoS	98.79	98.38	98.28	98.33	97.38
R2L	99.68	80.92	77.68	79.27	79.12
Probe	98.91	93.02	95.43	94.21	93.62
U2R	99.96	00.00	00.00	00.00	00.00
Normal	99.34	99.51	99.26	99.38	98.67
Average	99.34	74.37	74.13	74.24	73.76
TSS (30%)					
DoS	98.79	98.42	98.30	98.36	97.39
R2L	99.60	74.91	73.65	74.28	74.08
Probe	98.94	93.34	95.22	94.27	93.69
U2R	99.96	00.00	00.00	00.00	00.00
Normal	99.25	99.38	99.22	99.30	98.50
Average	99.31	73.21	73.28	73.24	72.73

**Table 4:** Comparative analysis of the outcomes of the MOSOA-DLVD algorithm and other algorithms [26,27,28].

Methods	$Accu_{y}$	$Prec_{n}$	$Reca_{l}$	$F_{Score}$
MOSOA-DLVD	99.34	74.37	74.13	74.24
LKM-OFLS [22]	89.34	64.64	54.68	58.26
K-Means-OFLS [23]	91.43	65.74	55.51	58.33
MLP algorithm [23]	91.46	66.61	56.76	54.99
PCA-NN [24]	90.08	64.56	56.06	57.54

## Data Availability

Data are contained within the article.
